# Radiation-induced magnetoresistance oscillations in monolayer and bilayer graphene

**DOI:** 10.1038/s41598-019-43866-4

**Published:** 2019-05-13

**Authors:** R. G. Mani, A. Kriisa, R. Munasinghe

**Affiliations:** Georgia State University, Department of Physics and Astronomy, Atlanta, GA 30303 USA

**Keywords:** Two-dimensional materials, Characterization and analytical techniques, Electronic properties and materials

## Abstract

We examine the characteristics of the microwave/mm-wave/terahertz radiation-induced magnetoresistance oscillations in monolayer and bilayer graphene and report that the oscillation frequency of the radiation-induced magnetoresistance oscillations in the massless, linearly dispersed monolayer graphene system should depend strongly both on the Fermi energy, and the radiation frequency, unlike in the case of the massive, parabolic, GaAs/AlGaAs 2D electron system, where the radiation-induced magnetoresistance oscillation frequency depends mainly on the radiation frequency. This possible dependence of the magnetoresistance oscillation frequency on the Fermi level at a fixed radiation frequency also suggests a sensitivity to the gate voltage in gated graphene, which suggests an *in*-*situ* tunable photo-excitation response in monolayer graphene that could be useful for sensing applications. In sharp contrast to monolayer graphene, bilayer graphene is expected to show radiation-induced magnetoresistance oscillations more similar to the results observed in the GaAs/AlGaAs 2D system. Such expectations for the radiation-induced magnetoresistance oscillations are presented here to guide future experimental studies in both of these modern atomic layer material systems.

## Introduction

Carrier scattering gives rise to electrical resistance – a measure of frictional losses within semiconductor specimens^1^. Under microwave/mm-wave/terahertz photoexcitation, high mobility 2D electron systems confined, for example, within GaAs/AlGaAs heterostructures exhibit large amplitude “1/4 cycle shifted” magnetoresistance oscillations with resistance maxima in the vicinity of *E* = (*j* + 3/4)*ħω*_*c*_, resistance minima near *E* = (*j* + 1/4)*ħω*_*c*_, and nodes in the resistance oscillations in the vicinity of the cyclotron resonance, and integral and half-integral cyclotron resonance harmonics, i.e., *E* = *jħω*_*c*_ and *E* = (*j* + 1/2)*ħω*_*c*_. Here, E = energy, *ω*_*c*_ = *eB*/m*, *e* = electron charge, m* = electron effective mass, *ħ* = the reduced Planck constant, and *B* = the magnetic field. Most remarkably, at the lowest temperatures under modest photo-excitation, the deepest resistance minima saturate into zero-resistance states^2^, about *B* = 4/5*B*_*f*_ and *B* = 4/9*B*_*f*_, of the characteristic field *B*_*f*_ = 2πfm*/*e*, where *f* is the electromagnetic-wave frequency^2–83^. That is, experiments suggest that the diagonal resistance can be switched off in the 2D electron system by photo-excitation in the presence of a small magnetic field. Such experimental reports have motivated the theoretical study of transport in photo-excited 2D semiconductor specimens^57–83^. Large amplitude magnetoresistance oscillations under photoexcitation also imply strong electrical sensitivity to electromagnetic waves, which could potentially be applied towards the realization of microwave/mm-wave/terahertz photodetectors^84–87^. Indeed, since the observations thus far have indicated *B*_*f*_ = 2πfm*/*e*, the implication is that radiation frequency *f* = *eB*_*f*_/2πm* can be determined from the characteristic field *B*_*f*_ of the magnetoresistance oscillations. The concurrent observed sensitivity to both the radiation-intensity and frequency has suggested the possibility of a tuned narrow band radiation sensor in the microwave/mm-wave/terahertz bands^25^.

Above mentioned radiation-induced magnetoresistance oscillations have already served to characterize material systems such as GaAs/AlGaAs heterostructures^2,3,5–7,9,11,13,15–17,19–55^, strained Si/SiGe^88^, electrons on the surface of liquid Helium^44,89^, and the oxide MgZnO/ZnO 2DES system^90^ for scattering lifetimes, and effective masses. Thus, one expects that photo-excited magnetotransport studies of modern atomic layer 2D systems such as monolayer and bilayer graphene could potentially unveil new science and applications^46,91–99^. However, the expectations for device response of these materials is not known and such phenomena have not been observed thus far in the graphene system. As a consequence, a question of interest is: what will be the oscillatory resistance response of modern atomic layered 2D materials such as, for example, graphene under electromagnetic wave excitation in a magnetic field? Below, we examine the expected characteristics for both the monolayer and bilayer graphene systems. As mentioned above, our results suggest a strong sensitivity of the response in monolayer graphene to both the Fermi energy and the radiation frequency. However, bilayer graphene is expected to show a more GaAs/AlGaAs like behavior, characteristic of parabolically dispersed systems. The results are summarized below.

It is well known that the Landau level dispersion for Dirac fermions in monolayer graphene is given by *E*_*N*_ = *sgn*(*N*)(2*ħeBN*)^1/2^*v*_*F*_^97,98^. Here, *N* is the Landau level index, and *v*_*F*_ is the carrier velocity. Suppose that the Fermi level lies in the *N*th Landau level, *E*_*N*_ = *E*_*F*_, and the electromagnetic radiation induces transitions from *N*’th Landau level to the *N* + *q*’th Landau level^57,59–61^, such that *E*_*N*_ + *q* = *E*_*F*_ + *hf*, where *h* is Planck’s constant, and *f* is the radiation frequency. From studies of the GaAs/AlGaAs system^2^, it is known that nodes in the radiation-induced magnetoresistance oscillations appear when the radiation energy spans such an integral number of Landau levels, i.e., *E*_*N*+*q*_ − *E*_*N*_ = *hf*, where *q* denotes the order of the node. Consider ${E}_{N}^{2}=2\hslash eBN{v}_{F}^{2}$, and note that

1 $${E}_{N+q}^{2}-{E}_{N}^{2}=2\hslash eBq{v}_{F}^{2}$$

Apply the identity ${E}_{N+q}^{2}-{E}_{N}^{2}=({E}_{N+q}-{E}_{N})({E}_{N+q}+{E}_{N})$. Let *E*_*N*+*q*_ − *E*_*N*_ = *hf* and *E*_*N*+*q*_ + *E*_*N*_ = 2*E*_*F*_ + *hf*, Then, by substituting into Eq. 1, we obtain

2 $$(2{E}_{F}+hf)hf=2\hslash eBq{v}_{F}^{2}$$

or

3 $$B=(1/q)(\pi f/e)(2{E}_{F}/{v}_{F}^{2}+hf/{v}_{F}^{2})$$

**Figure 1:**
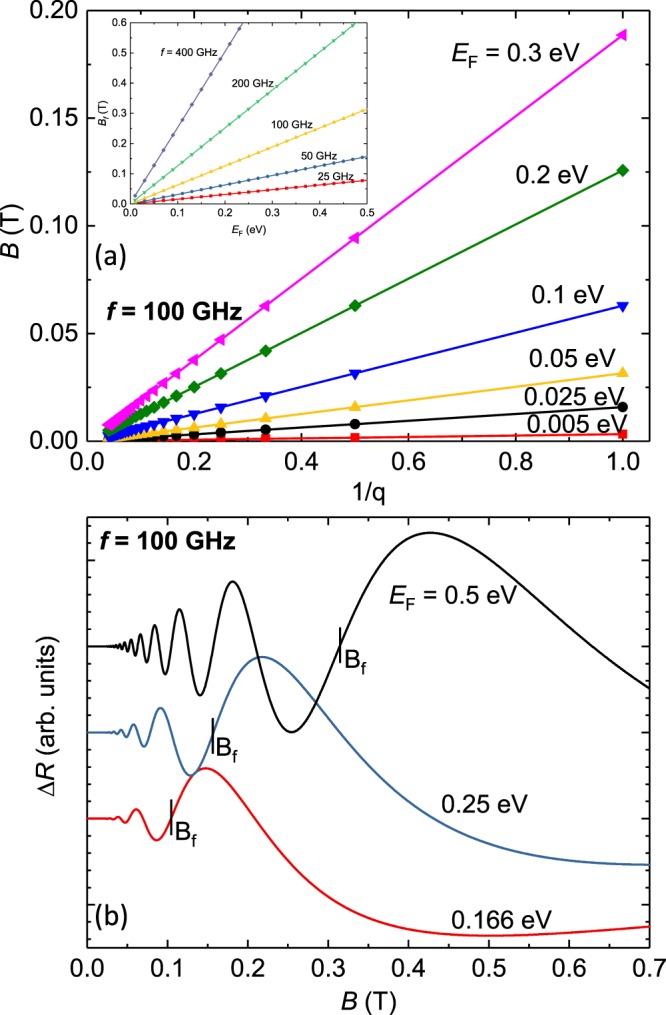
(**a**) For monolayer graphene, the magnetic field values, *B*, for nodes in the microwave induced magnetoresistance oscillations are plotted vs. the inverse node index, 1/*q*, with *q* = 1, 2, 3…, for different values of the Fermi energy, *E*_*F*_, at a microwave frequency of *f* = 100 GHz. The inset shows the characteristic field or oscillation frequency *B*_*f*_ of the microwave induced magnetoresistance oscillations as a function of the Fermi energy, *E*_*F*_, for radiation frequencies *f* = 25, 50, 100, 200, and 400 GHz. (**b**) This figure illustrates the expected oscillatory magnetoresistance, Δ*R*, vs. *B*, in monolayer graphene under microwave photo-excitation at *f* = 100 GHz for three values of the Fermi energy, *E*_*F*_. Note that the characteristic magnetic field, *B*_*f*_, of the oscillatory magneto-resistance increases with *E*_*F*_.

Thus, Eq. 3 describes the magnetic field values for the *q*’th node of radiation-induced magnetoresistance oscillations in monolayer graphene and it suggests that such oscillations in monolayer graphene are also dependent upon value of the Fermi energy, *E*_*F*_, unlike in the GaAs/AlGaAs system^2,5,7,9,11,13,15,17,25,27,31,35,41^. In Fig. 1(a), we plot the nodal positions in magnetic field, *B*, vs. the inverse of integers, 1/*q*, for several typical values of the Fermi energy in monolayer graphene. The figure shows a set of straight lines, which indicates that the expected magnetoresistance oscillations are periodic in *B*^−1^, just as in the GaAs/AlGaAs system. The characteristic frequency or field, *B*_*f*_, of the radiation-induced magnetoresistance oscillations that appears in the empirical formula for the oscillatory magnetoresistance lineshape Δ*R* ≈ −*exp*(−*λ*/*B*) *sin* (2*πB*_*f*_/*B*)^11^, is found from the slopes in Fig. 1(a) of *B* vs. 1/*q*, which suggest increases of *B*_*f*_ with *E*_*F*_, at a fixed radiation frequency, *f* = 100 GHz. The inset of Fig. 1(a) shows the variation of this characteristic frequency *B*_*f*_ of the radiation-induced magnetoresistance oscillation with *E*_*F*_ at several radiation frequencies, *f* = 25, 50 100, 200 and 400 GHz. The inset conveys that *B*_*f*_ increases faster with *E*_*F*_ at larger radiation frequencies *f*. Note that *B*_*f*_ increases linearly with *f* over this range of *f* since *E*_*F*_ ≫ *hf*, see eqn. 3. For *f* = 100 GHz, *hf* = 4.125 × 10^−4^ eV is much smaller than a small practical value for *E*_*F*_ such as *E*_*F*_ = 5 × 10^−3^ eV.

The expectations for the oscillatory resistance, Δ*R* ≈ −*exp*(−*λ*/*B*) *sin* (2*πB*_*f*_/*B*)^11^, in the monolayer graphene system are illustrated in Fig. 1(b), which exhibits Δ*R* vs. *B* at *E*_*F*_ = 0.5, 0.25, and 0.166 eV for photoexcitation at *f* = 100 GHz. Since the characteristic field *B*_*f*_ increases with *E*_*F*_, and the parameters *B*, *f*, *e*, and *v*_*F*_ in Eq. 3 can be determined or are well known, the radiation-induced magnetoresistance oscillations in monolayer graphene can be utilized to accurately determine *E*_*F*_.

From the experimental perspective, measurements can be carried out as a function of the magnetic field, *B*, or, as is more typical for graphene, vs. the gate voltage *V*_*G*_. In monolayer graphene, the carrier density, *n*_*q*_, varies as the square of the Fermi energy, i.e., *n*_*q*_ = (4*π*/*h*^2^)(*E*_*F*_/*v*_*F*_)^2^. Then,

4 $${B}_{f}=(\pi f/e)(1/{v}_{F}^{2})(2h{v}_{F}{({n}_{q}/4\pi )}^{1/2}+hf)$$

Further, for graphene on top of 300 nm *SiO*_2_ on doped *Si* the relation between the gate voltage and the carrier density is *n*_*q*_ = *sgn*(Δ*V*_*G*_)*α*|Δ*V*_*G*_| with *α* = 7.2 × 10^10^ *cm*^2^/*V*^97,98^. Upon inserting these relations, we obtain from eqn. 4:

5 $${B}_{f}=(\pi f/e)(1/{v}_{F}^{2})(2h{v}_{F}{(\alpha |{\rm{\Delta }}{V}_{G}|/4\pi )}^{1/2}+hf)$$

**Figure 2:**
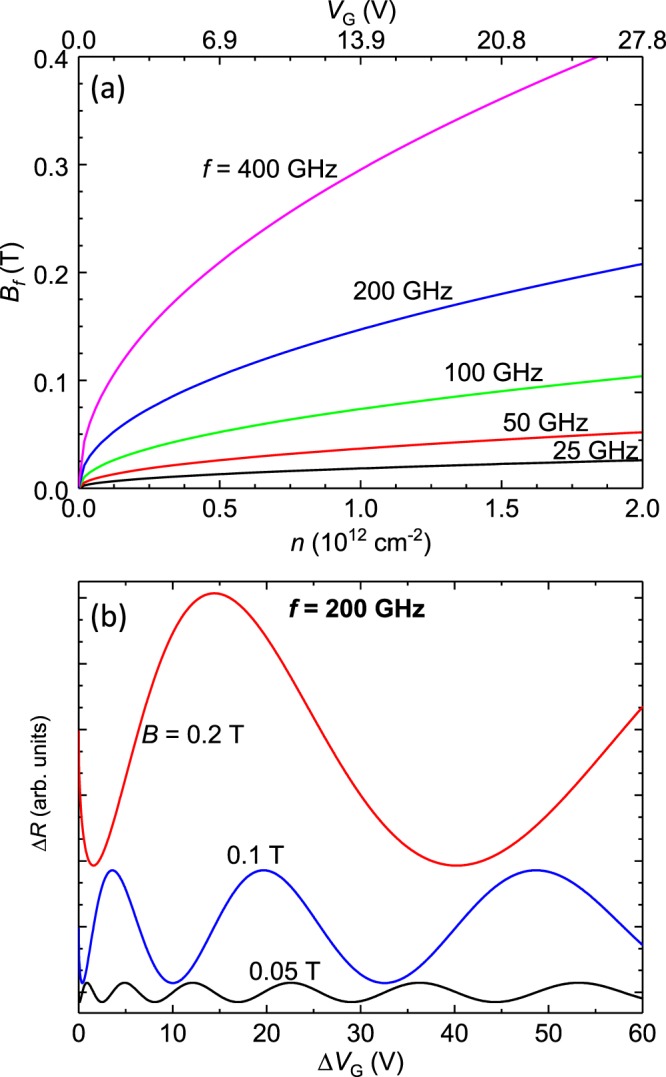
(**a**) The characteristic field or oscillation frequency *B*_*f*_ of radiation-induced magneto-resistance oscillations in monolayer graphene is plotted vs. the electron density, *n*, (bottom abscissa), and the gate voltage, Δ*V*_*G*_, (top abscissa). *B*_*f*_ is proportional to *n*^1/2^ and ${\rm{\Delta }}{V}_{G}^{1/2}$. (**b**) This figure illustrates the expected oscillatory magneto-resistance, Δ*R*, vs. the gate voltage difference with respect to the neutrality voltage, Δ*V*_*G*_, in monolayer graphene under microwave photo-excitation at *f* = 200 GHz for three values of the magnetic field, *B*. Here, since the extrema are periodic in (Δ*V*_*G*_)^1/2^, they become further apart with increasing Δ*V*_*G*_.

Figure 2(a) exhibits the dependence of the frequency or characteristic field *B*_*f*_ of the magnetoresistance oscillations vs. the electron density, i.e., *n*_*q*_ = *n*, and vs. the difference in the gate voltage with respect to the neutrality voltage, *V*_*N*_, i.e., Δ*V*_*G*_ = *V*_*G*_ − *V*_*N*_. The square root dependence observed in Eqs 4 and 5 is manifested as a sub-linear variation of *B*_*F*_ with respect to these parameters at a fixed radiation frequency, *f*, i.e., *B*_*f*_ ≈ *n*^1/2^ and ${B}_{f}\approx {\rm{\Delta }}{V}_{G}^{1/2}$. Expectations for gate voltage dependence of the radiation-induced magnetoresistance oscillations in monolayer graphene following from Eq. 5 are exhibited in Fig. 2(b) for *f* = 200 GHz. The Fig. 2(b) shows that oscillations grow in amplitude with increasing magnetic field. Further, it is evident that the number of oscillations over a given span of gate voltage decreases with increasing *B*. Finally, the spacing between, say, successive oscillatory minima, increases with increasing Δ*V*_*G*_ because the magnetoresistance oscillations are actually periodic in ${\rm{\Delta }}{V}_{G}^{1/2}$. From these numerical studies, it is clear that radiation-induced oscillations in monolayer graphene should be substantially different than the oscillations observed in the GaAs/AlGaAs system. The key differences are the dependence of the characteristic field or frequency *B*_*f*_ of the magnetoresistance oscillations upon the Fermi energy when *E*_*F*_ ≫ *hf* even at a fixed radiation frequency in monolayer graphene (Fig. 1(a)), the expected dependence of *B*_*f*_ on the gate voltage (Fig. 2), and the possibility of using such magnetoresistance oscillations to make a measurement of the Fermi energy in monolayer graphene.

Unlike in monolayer graphene, charge carriers in bilayer graphene are massive fermions as a consequence of the parabolic band structure. Bilayer graphene is zerogap system in the absence of a transverse electric field and it develops a bandgap under a transverse electric field^97,98^. Consider the zerogap case: The dispersion of carriers in zerogap bilayer graphene under the influence of a magnetic field is given by *E*_*N*_ = (*N*(*N* ± 1))^1/2^*ω*_0_*B*, where *ω*_0_ = *eħ*/m*, and m* = 0.037 *m* is the nominal effective mass of the carriers^97,98^. Here, the + and − describe the response of the electron and the hole systems, and *N* can take on positive and negative integers for electrons and holes, respectively. To determine the expected response for the radiation-induced magnetoresistance oscillations in bilayer graphene with electrons, we suppose that at a magnetic field, *B*, the Fermi level lies in the *N*th Landau level, *E*_*N*_ = *E*_*F*_, and the electromagnetic radiation induces transition from Landau level *N* to the *N* + *q* Landau level so that *E*_*N*+*q*_ = *E*_*F*_ + *hf*. Further, assume that nodes in the radiation-induced magnetoresistance oscillations will appear when the radiation energy spans an integral number of Landau levels, i.e., *E*_*N*+*q*_ − *E*_*q*_ = *hf*, where *q* denotes the order of the node. To extract the characteristics for radiation-induced magnetoresistance oscillations in such a system, we examine:

6 $${E}_{N+q}^{2}-{E}_{N}^{2}=(hf)(2{E}_{F}+hf)={\omega }_{0}^{2}{B}^{2}q(q+2N+1)$$

By setting ${E}_{N}^{2}={E}_{F}^{2}=N(N+1){\omega }_{0}^{2}{B}^{2}$, we find $(N+1/2)={(({E}_{F}^{2}/{\omega }_{0}^{2}{B}^{2})+1/4)}^{1/2}$, to obtain

7 $$q={[hf(2{E}_{F}+hf)/({\omega }_{0}^{2}{B}^{2})+({E}_{F}^{2}/({\omega }_{0}^{2}{B}^{2})+1/4)]}^{1/2}-{({E}_{F}^{2}/({\omega }_{0}^{2}{B}^{2})+1/4)}^{1/2}$$

**Figure 3:**
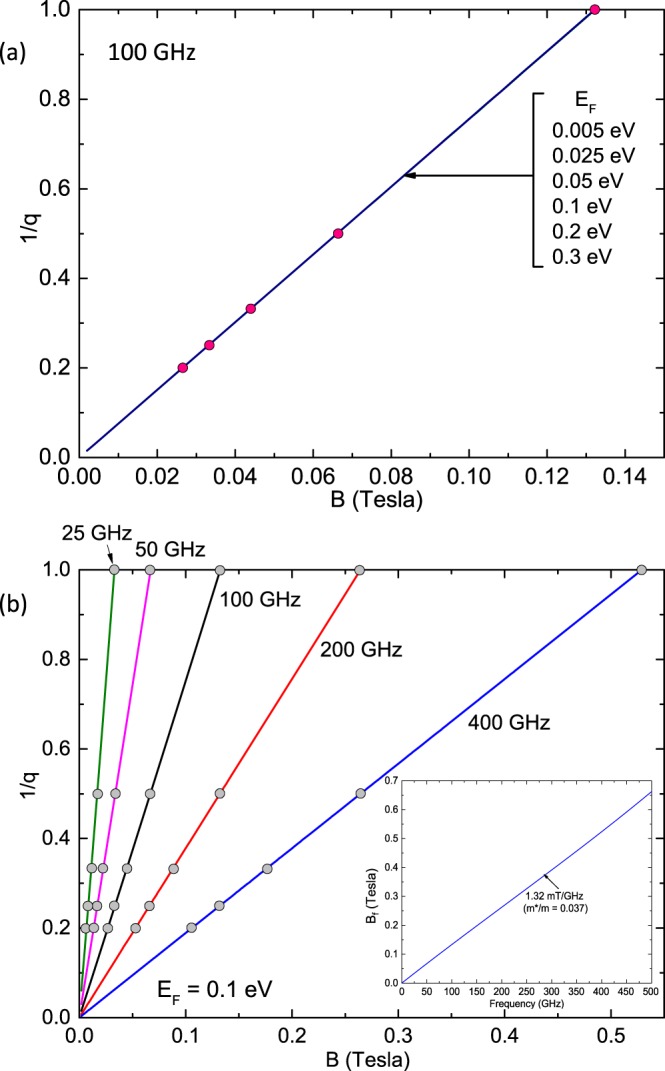
(**a**) For radiation-induced magneto-resistance oscillations in bilayer graphene, the inverse node index, 1/*q*, for *q* = 1, 2, 3… is plotted vs the magnetic field, *B*, for various Fermi energies, *E*_*F*_, at a radiation frequency, *f* = 100 GHz. (**b**) For radiation-induced magnetoresistance oscillations in bilayer graphene, the inverse node index, 1/*q*, for *q* = 1, 2, 3… is plotted vs. the magnetic field, *B*, for radiation frequencies *f* = 25, 50, 100, 200, and 400 GHz. The plot implies that increasing the radiation frequency shifts the magnetoresistance oscillations to higher magnetic fields. The inset shows the magnetoresistance oscillation frequency, *B*_*f*_, vs the radiation frequency, *f*. The plot shows that the slope of the line is 1.32 mT/GHz which corresponds to an effective mass ratio m*/*m* = 0.037.

Equation 7 serves to determine the B-position of the nodes in the radiation-induced magnetoresistance oscillations as a function of the magnetic field for different values of the Fermi energy, *E*_*F*_. Figure 3(a) presents 1/*q* vs. *B* for different values of the *E*_*F*_ at *f* = 100 GHz for bilayer graphene. The remarkable feature in this figure is the relative insensitivity of the results to the value of the Fermi energy for bilayer graphene, unlike in the case of monolayer graphene (see Fig. 1(a)). Thus, in this sense, bilayer graphene looks more similar to the GaAs/AlGaAs system.

The expected nodal *B*-positions, which are insensitive to *E*_*F*_ in bilayer graphene, are examined for different *f* in Fig. 3(b). Figure 3(b) shows that a given node, *q* = 1, 2, 3…, shifts to higher *B* as *f* increases. Further, the frequency or characteristic field *B*_*f*_ of the radiation-induced magneto-resistance oscillations increases linearly with *f* in the limit where *E*_*F*_ ≫ *hf*, as indicated in the inset of Fig. 3(b). Indeed, the characteristic field or of frequency *B*_*f*_-field should shift at the rate of 1.37 mT/GHz in bilayer graphene vis-à-vis the ≈2.35 mT/GHz shift observed in the GaAs/AlGaAs system. These calculations have assumed a fixed effective mass of m* = 0.037 *m* for bilayer graphene, while it is known that non-parabolicity could provide for variation in m* with *E*_*F*_^97,98^. Thus, studies of radiation induced magnetoresistance oscillations in bilayer graphene as a function of the carrier density could serve to characterize the non-parabolicity and determine the effective mass as a function of the energy.

In comparing the expectations for monolayer and bilayer graphene, the striking feature is the great dissimilarity in the *E*_*F*_ dependence of the radiation-induced oscillatory magnetoresistance characteristics. For monolayer graphene, the oscillations depend strongly on *E*_*F*_ and therefore also the Δ*V*_*G*_. On the other hand, for bilayer graphene, there is a lack of sensitivity to *E*_*F*_ and therefore also on Δ*V*_*G*_. This suggests that the characteristics of the observed magnetoresistance oscillations could also serve to differentiate between these two types of graphene.

## References

[1] Ando T, Fowler AB, Stern F (1982). Electronic properties of two-dimensional systems. Rev. Mod. Phys..

[2] Mani RG (2002). Zero-resistance states induced by electromagnetic wave excitation in GaAs/AlGaAs heterostructures. Nature.

[3] Zudov MA, Du RR, Pfeiffer LN, West KW (2003). Evidence for a new dissipationless effect in 2D electronic transport. Phys. Rev. Lett..

[4] Fitzgerald R (2003). Microwaves induce vanishing resistance in two dimensional electron systems. Phys. Today.

[5] Mani, R. G. *et al*. Demonstration of a 1/4 cycle phase shift in the radiation-induced oscillatory-magnetoresistance in GaAs/AlGaAs devices. *Phys*. *Rev*. *Lett*. **92**, 146801-1-4 (2004). 10.1103/PhysRevLett.92.146801 15089564

[6] Kovalev AE, Zvyagin SA, Bowers CR, Reno JL, Simmons JA (2004). Observation of a node in the quantum oscillations induced by microwave radiation. Sol. St. Comm..

[7] Mani, R. G. *et al*. Radiation induced oscillatory Hall effect in high mobility GaAs/AlGaAs devices. *Phys*. *Rev*. *B* **69**, 161306-1-4 (2004).

[8] Dorozhkin SI (2003). Giant magnetoresistance oscillations caused by cyclotron resonance harmonics. JETP letters.

[9] Mani, R. G. *et al*. Radiation induced zero-resistance states in GaAs/AlGaAs heterostructures: Voltage-current characteristics and intensity dependence at the resistance minima. *Phys*. *Rev*. *B* **70**, 155310-1-5 (2004).

[10] Zehnder, C., Wirthmann, A., Heyn, C. & Heitmann, D. Bolometric spin effect due to internal spin injection in a two dimensional electron system. *EuroPhys*. *Lett*., textbf63, 576–582 (2003).

[11] Mani, R. G. *et al*. Radiation-induced oscillatory magnetoresistance as a sensitive probe of the zero-field spin splitting in high-mobility GaAs/AlGaAs devices. *Phys*. *Rev*. *B* **69**, 193304-1-4 (2004).

[12] Studenikin SA, Potemski M, Coleridge PT, Sachrajda AS, Wasilewski ZR (2004). Microwave radiation induced magneto-oscillation in the longitudinal and transverse resistance of a two dimensional electron gas. Sol. St. Comm..

[13] Mani RG (2004). Zero-resistance states induced by electromagnetic-wave excitation in GaAs/AlGaAs heterostructures. Physica E (Amsterdam).

[14] Du RR, Zudov MA, Yang CL, Pfeiffer LN, West KW (2004). Dissipationless 2D electronic transport effect induced by microwaves. Physica E (Amsterdam).

[15] Mani RG (2004). Radiation-induced zero-resistance states with resolved Landau levels. Appl. Phys. Lett..

[16] Simovic, B., Ellenberger, C., Ensslin, K. & Wegscheider, W. Density dependence of microwave induced magnetoresistance oscillations in a two-dimensional electron gas. *Phys*. *Rev*. *B* **71**, 233303-1-4 (2005).

[17] Mani, R. G. Radiation-induced oscillatory magnetoresistance in a tilted magnetic field in GaAs/AlGaAs devices. *Phys*. *Rev*. *B* **72**, 075327-1-5 (2005). 10.1103/PhysRevLett.92.146801 15089564

[18] Kukushkin IV (2004). New type of B-periodic magneto-oscillations in a two dimensional electron system induced by microwave irradiation. Phys. Rev. Lett..

[19] Mani RG (2004). Photo-excited zero-resistance states in quasi-two-dimensional GaAs/AlGaAs devices. Sol. St. Comm..

[20] Smet, J. H. *et al*. Circular-polarization-dependent study of the microwave photoconductivity in a two-dimensional electron system. *Phys*. *Rev*. *Lett*. **95**, 116804-1-4 (2005). 10.1103/PhysRevLett.95.116804 16197030

[21] Mani RG (2005). Spin characterization and control over the regime of the radiation-induced zero-resistance states. IEEE Trans. Nanotechnol..

[22] Mani, R. G. Radiation-induced decay of Shubnikov-de Haas oscillations in the regime of the radiation-induced zero-resistance states. *Appl*. *Phys*. *Lett*. **91**, 132103-1-3 (2007).

[23] Wirthmann, A. *et al*. Far-infrared-induced magnetoresistance oscillations in GaAs/AlGaAs-based two-dimensional electron systems. *Phys*. *Rev*. *B* **76**, 195315-1-5 (2007).

[24] Studenikin, S. A. *et al*. Frequency quenching of microwave-induced resistance oscillations in a high-mobility two-dimensional electron gas. *Phys*. *Rev*. *B* **76**, 165321-1-6 (2007).

[25] Mani, R. G. Narrow-band radiation-sensing in the Terahertz and microwave bands using the radiation-induced magnetoresistance oscillations. *Appl*. *Phys*. *Lett*. **92**, 102107-1-3 (2008).

[26] Wiedmann, S. *et al*. Interference oscillations of microwave photoresistance in double quantum wells. *Phys*. *Rev*. *B* **78**, 121301-1-4 (2008).

[27] Mani, R. G., Johnson, W. B., Umansky, V., Narayanamurti, V. & Ploog, K. Phase study of oscillatory resistances in microwave irradiated and dark GaAs/AlGaAs devices: Indications of an unfamiliar class of integral quantum Hall effect. *Phys*. *Rev*. *B* **79**, 205320-1-10 (2009).

[28] Chepelianskii, A. D. & Shepelyansky, D. L. Microwave stabilization of edge transport and zero-resistance states. *Phys*. *Rev*. *B* **80**, 241308-1-4 (2009).

[29] Wiedmann, S. *et al*. Magnetoresistance oscillations in multilayer systems: Triple quantum wells. *Phys*. *Rev*. *B* **80**, 245306-1-9 (2009).

[30] Konstantinov, D. & Kono, K. Photon-induced vanishing of magnetoconductance in 2D electrons on liquid helium. *Phys*. *Rev*. *Lett*. **105**, 226801-1-4 (2010). 10.1103/PhysRevLett.105.226801 21231410

[31] Mani, R. G., Gerl, C., Schmult, S., Wegscheider, W. & Umansky, V. Nonlinear growth with the microwave intensity in the radiation-induced magnetoresistance oscillations. *Phys*. *Rev*. *B* **81**, 125320-1-6 (2010).

[32] Wiedmann, S., Gusev, G. M., Raichev, O. E., Bakarov, A. K. & Portal, J. C. Thermally activated intersubband scattering and oscillating magnetoresistance in quantum wells. *Phys*. *Rev*. *B* **82**, 165333-1-8 (2010).

[33] Wiedmann, S., Gusev, G. M., Raichev, O. E., Bakarov, A. K. & Portal, J. C. Microwave zero-resistance states in a bilayer electron system. *Phys*. *Rev*. *Lett*. **105**, 026804-1-4 (2010). 10.1103/PhysRevLett.105.026804 20867726

[34] Ramanayaka, A. N., Mani, R. G. & Wegscheider, W. Microwave induced electron heating in the regime of the radiation-induced magnetoresistance oscillations. *Phys*. *Rev*. *B* **83**, 165303-1-5 (2011).

[35] Mani, R. G., Ramanayaka, A. N. & Wegscheider, W. Observation of linear-polarization-sensitivity in the microwave-radiation-induced magnetoresistance oscillations. *Phys*. *Rev*. *B* **84**, 085308-1-4 (2011).

[36] Ramanayaka, A. N., Mani, R. G., Inarrea, J. & Wegscheider, W. Effect of rotation of the polarization of linearly polarized microwaves on the radiation-induced magnetoresistance oscillations. *Phys*. *Rev*. *B* **85**, 205315-1-6 (2012).

[37] Mani RG, Hankinson J, Berger C, de Heer WA (2012). Observation of resistively detected hole spin resonance and zero-field pseudo-spin splitting in graphene. Nature Commun..

[38] Konstantinov, D., Monarkha, Y. & Kono, K. Effect of coulomb interaction on microwave-induced magnetoconductivity oscillations of surface electrons on liquid helium. *Phys*. *Rev*. *Lett*. **111**, 266802-1-5 (2013). 10.1103/PhysRevLett.111.266802 24483809

[39] Mani RG, Kriisa A, Wegscheider W (2013). Magneto-transport characteristics of a 2D electron system driven to negative magneto-conductivity by microwave photoexcitation. Sci. Rep..

[40] Mani, R. G. *et al*. Terahertz photovoltaic detection of cyclotron resonance in the regime of the radiation-induced magnetoresistance oscillations. *Phys*.*Rev*. *B* **87**, 245308-1-8 (2013).

[41] Mani RG, Kriisa A, Wegscheider W (2013). Size-dependent giant-magnetoresistance in millimeter scale GaAs/AlGaAs 2D electron devices. Sci. Rep..

[42] Mani RG, von Klitzing K, Ploog K (1993). Magnetoresistance over the intermediate localization regime in GaAs/AlGaAs quantum wires. Phys. Rev. B.

[43] Ye, T., Liu, H-C., Wegscheider, W. & Mani, R. G. Combined study of microwave-power/linear polarization dependence of the microwave-radiation-induced magnetoresistance oscillations in GaAs/AlGaAs devices. *Phys*. *Rev*. *B* **89**, 155307-1-5 (2014).

[44] Chepelianskii AD, Watanabe N, Nasyedkin K, Kono K, Konstantinov D (2015). An incompressible state of a photo-excited electron gas. Nat. Comm..

[45] Ye T, Liu H-C, Wang Z, Wegscheider W, Mani RG (2015). Comparative study of microwave radiation-induced magnetoresistive oscillations induced by circularly- and linearly- polarized photoexcitation. Sci. Rep..

[46] Mani RG (2016). Method for determining the residual electron- and hole- densities about the neutrality point over the gate-controlled n-p transition in graphene. Appl. Phys. Lett..

[47] Herrmann, T. *et al*. Analog of microwave-induced resistance oscillations induced in GaAs heterostructures by terahertz radiation. *Phys*. *Rev*. *B* **94**, 081301-1-5 (2016).

[48] Liu, H-C., Samaraweera, R. L., Reichl, C., Wegscheider, W. & Mani, R. G. Study of the angular phase shift in the polarization angle dependence of the microwave induced magnetoresistance oscillations. *Phys*. *Rev*. *B* **94**, 245312-1-7 (2016).

[49] Shi Q (2015). Shubnikov de Haas oscillations in a two-dimensional electron gas under subterahertz radiation. Phys. Rev. B.

[50] Wang Z, Samaraweera RL, Reichl C, Wegscheider W, Mani RG (2016). Tunable electron heating induced giant magnetoresistance in the high mobility GaAs/AlGaAs 2D electron system. Sci. Rep..

[51] Samaraweera RL (2017). Mutual influence between current-induced giant magnetoresistance and radiation-induced magnetoresistance oscillations in the GaAs/AlGaAs 2DES. Sci. Rep..

[52] Liu H-C, Reichl C, Wegscheider W, Mani RG (2018). B-periodic oscillations in the Hall resistance induced by a dc-current bias under combined microwave excitation and dc-current bias in the GaAs/AlGaAs 2D system. Scientific Reports.

[53] Samaraweera RL (2018). Coherent backscattering in the quasi-ballistic ultra-high mobility GaAs/AlGaAs 2DES. Scientific Reports.

[54] Munasinghe CR (2018). Electron heating induced by an ac-bias current in the regime of Shubnikov-de Haas oscillations in the high mobility GaAs/AlGaAs two-dimensional electron system. J Phys Condens Matter.

[55] Nanayakkara TR (2018). Electron heating induced by microwave photoexcitation in the GaAs/AlGaAs two-dimensional electron system. Phys. Rev. B..

[56] Mani RG, von Klitzing K (1992). Localization at high magnetic fields in GaAs/AlGaAs. Phys. Rev. B.

[57] Durst, A. C., Sachdev, S., Read, N. & Girvin, S. M. Radiation-induced magnetoresistance oscillations in a 2D electron gas. *Phys*. *Rev*. *Lett*. **91**, 086803-1-4 (2003). 10.1103/PhysRevLett.91.086803 14525267

[58] Ryzhii V, Suris R (2003). Nonlinear effects in microwave photoconductivity of two-dimensional electron systems. J. Phys.: Cond. Matt..

[59] Shi J, Xie XC (2003). Radiation-induced zero-resistance state and the photon-assisted transport. Phys. Rev. Lett..

[60] Ryzhii VI (2003). Absolute negative conductivity in two-dimensional electron systems associated with acoustic scattering stimulated by microwave radiation. Phys. Rev. B.

[61] Lei XL, Liu SY (2003). Radiation-induced magnetoresistance oscillation in a two-dimensional electron gas in Faraday geometry. Phys. Rev. Lett..

[62] Andreev, A. V., Aleiner, I. L. & Millis, A. J. Dynamical symmetry breaking as the origin of the zero-dc-resistance state in an ac-driven system. *Phys*. *Rev*. *Lett*. **91**, 056803-1-4 (2003). 10.1103/PhysRevLett.91.056803 12906622

[63] Koulakov, A. A. & Raikh, M. E. Classical model for the negative dc conductivity of ac-driven two-dimensional electrons near the cyclotron resonance. *Phys*. *Rev*. *B* **68**, 115324-1-4 (2003).

[64] Lei, X. L. & Liu, S. Y. Radiation-induced magnetoresistance oscillation in a two-dimensional electron gas in Faraday geometry. *Phys*. *Rev*. *Lett*. **91**, 226805-1-4 (2003). 10.1103/PhysRevLett.91.226805 14683265

[65] Rivera, P. H. & Schulz, P. A. Radiation-induced zero-resistance states: Possible dressed electronic structure effects. *Phys*. *Rev*. *B* **70**, 075314-1-6 (2004).

[66] Mikhailov SA (2004). Microwave-induced magnetotransport phenomena in two-dimensional electron systems: Importance of electrodynamic effects. Phys. Rev. B..

[67] Dmitriev, I. A., Vavilov, M. G., Aleiner, I. L., Mirlin, A. D. & Polyakov, D. G. Theory of microwave-induced oscillations in the magnetoconductivity of a two-dimensional electron gas. *Phys*. *Rev*. *B* **71**, 115316-1-11 (2005).

[68] Torres, M. & Kunold, A. Kubo formula for Floquet states and photoconductivity oscillations in a two-dimensional electron gas. *Phys*. *Rev*. *B* **71**, 115313-1-13 (2005).

[69] Lei, X. L. & Liu, S. Y. Radiation-induced magnetotransport in high mobility two-dimensional systems: Role of electron heating. *Phys*. *Rev*. *B* **72**, 075345-1-10 (2005).

[70] Inarrea, J. & Platero, G. Theoretical approach to microwave radiation-induced zero-resistance states in 2D electron systems. *Phys*. *Rev*. *Lett*. **94**, 016806-1-4 (2005). 10.1103/PhysRevLett.94.016806 15698116

[71] Inarrea, J. & Platero, G. Temperature effects on microwave-induced resistivity oscillations and zero-resistance states in two-dimensional electron systems. *Phys*. *Rev*. *B* **72**, 193414-1-4 (2005).

[72] Raichev, O. E. Magnetic oscillations of resistivity and absorption of radiation in quantum wells with two populated subbands. *Phys*. *Rev*. *B* **78**, 125304-1-14 (2008).

[73] Inarrea, J. Effect of frequency and temperature on microwave-induced magnetoresistance oscillations in two-dimensional electron systems. *Appl*. *Phys*. *lett*. **92**, 192113-1-3 (2008).

[74] Dmitriev, I. A., Khodas, M., Mirlin, A. D., Polyakov, D. G. & Vavilov, M. G. Mechanisms of the microwave photoconductivity in two-dimensional electron systems with mixed disorder. *Phys*. *Rev*. *B* **80**, 165327-1-9 (2009).

[75] Inarrea, J., Mani, R. G. & Wegscheider, W. Sublinear radiation power dependence of photoexcited resistance oscillations in two-dimensional electron systems. *Phys*. *Rev*. *B* **82**, 205321-1-5 (2010).

[76] Mikhailov, S. A. Theory of microwave-induced zero-resistance states in two-dimensional electron systems. *Phys*. *Rev*. *B* **83**, 155303-1-12 (2011).

[77] Inarrea, J. Influence of linearly polarized radiation on magnetoresistance in irradiated two-dimensional electron systems. *Appl*. *Phys*. *Lett*. **100**, 242103-1-3 (2012).

[78] Lei X. L. & Liu, S. Y. Linear polarization dependence of microwave-induced magnetoresistance oscillations in high mobility two-dimensional systems. *Phys*. *Rev*. *B* **86**, 205303-1-5 (2012).

[79] Iñarrea, J. Linear polarization sensitivity of magnetotransport in irradiated two-dimensional electron systems. *J*. *Appl*. *Phys*. **113**, 183717-1-5 (2013).

[80] Zhirov, O. V., Chepelianskii, A. D. & Shepelyansky, D. L. Towards a synchronization theory of microwave-induced zero-resistance states. *Phys*. *Rev*. *B* **88**, 035410-1-14 (2013).

[81] Raichev, O. E. Theory of magnetothermoelectric phenomena in high-mobility two-dimensional electron systems under microwave irradiation. *Phys*. *Rev*. *B* **91**, 235307-1-16 (2015).

[82] Beltukov, Y. M. & Dyakonov, M. I. Microwave-induced resistance oscillations as a classical memory effect. *Phys*. *Rev*. *Lett*. **116**, 176801-1-5 (2016). 10.1103/PhysRevLett.116.176801 27176530

[83] Chang C-C, Chen G-Y, Lin L (2016). Dressed photon induced resistance oscillation and zero-resistance in arrayed simple harmonic oscillators with no impurity. Sci. Rep..

[84] Phillips TG, Keene JS (1992). Astronomy. Proc. IEEE.

[85] Hu BB, Nuss MC (1999). Imaging with terahertz waves. Opt. Lett..

[86] Jacobsen RH, Mittleman DM, Nuss MC (1996). Chemical recognition of gases and gas mixtures with terahertz waves. Opt. Lett..

[87] Tonouchi M (2007). Cutting-edge terahertz technology. Nature Photonics.

[88] Zudov MA (2014). Observation of microwave-induced resistance oscillations in a high mobility two-dimensional hole gas in a strained Ge/SiGe quantum well. Phys. Rev. B.

[89] Yamashiro R, Abdurakhimov LV, Badrutinov AO, Monarkha YP, Konstantinov D (2015). Photoconductivity response at cyclotron-resonance harmonics in a nondegenerate two-dimensional electron gas on liquid Helium. Phys. Rev. Lett..

[90] Karcher DF (2016). Observation of microwave induced resistance and photovoltage oscillations in MgZnO/ZnO heterostructures. Phys. Rev. B.

[91] Novoselov KS (2004). Electric field effect in atomically thin carbon films. Science.

[92] Berger C (2004). Ultrathin epitaxial graphite: 2D electron gas properties and a route toward graphene-based nanoelectronics. J. Phys. Chem. B.

[93] Novoselov KS (2005). Two-dimensional gas of massless Dirac fermions in graphene. Nature.

[94] Zhang YB, Tan YW, Stormer HL, Kim P (2005). Experimental observation of the quantum Hall effect and Berry’s phase in graphene. Nature.

[95] Berger C (2006). Electronic confinement and coherence in patterned epitaxial graphene. Science.

[96] Geim AK, Novoselov KS (2007). The rise of graphene. Nature Mater..

[97] Castro Neto AH, Guinea F, Peres NMR, Novoselov KS, Geim AK (2009). The electronic properties of graphene. Rev. Mod. Phys..

[98] Das Sarma S, Adam S, Hwang EH, Rossi E (2011). Electronic transport in two-dimensional graphene. Rev. Mod. Phys..

[99] Witowski, A. M. *et al*. Quasiclassical cyclotron resonance of Dirac fermions in highly doped graphene. *Phys*. *Rev*. *B*, **82**, 165305-1-6 (2010).

